# Factors and Determinants of Choosing Pathology as a Future Career: Results From a Multi-Institution Study

**DOI:** 10.7759/cureus.15790

**Published:** 2021-06-21

**Authors:** Emad M Masuadi, Mohamud S Mohamud, Abdulrahman M Alhassan, Khalid G Alharbi, Ahmed S Hilabi, Faisal A Alharbi, Abdullah T Tatwani, Abdullah I Farraj, Sami Al-Nasser, Mohammed F Safi

**Affiliations:** 1 Research Unit/Biostatistics, King Saud Bin Abdulaziz University for Health Sciences/King Abdullah International Medical Research Centre (KAIMRC), Riyadh, SAU; 2 Medical Education, College of Medicine/King Saud Bin Abdulaziz University for Health Sciences, Riyadh, SAU; 3 Pathology and Laboratory Medicine, College of Applied Medical Sciences/King Saud University, Riyadh, SAU; 4 Medical Education, King Saud Bin Abdulaziz University for Health Sciences, Riyadh, SAU; 5 Pathology, United Arab Emirates University, Al Ain, ARE

**Keywords:** medical students, factors, career choice, pathology, perception

## Abstract

Background

Globally, less than 10% of graduating medical students select pathology as a future career. Many factors were reported from different settings to influence the choice of pathology. The aim of this study was to investigate the factors that determine medical students’ preferences in choosing pathology as a future career.

Methods

This cross-sectional study surveyed students from three governmental medical schools in Riyadh, Saudi Arabia. A self-administered questionnaire that contained demographic questions and items that addressed perceived factors that affect the choice of pathology was distributed to medical students. Collected data were analyzed using Statistical Product and Service Solutions (SPSS) Statistics for Windows, Version 25.0 (Armonk, NY: IBM Corp). A chi-square test was used to determine the association between independent variables and interest in pathology.

Results

Out of the 400 questionnaires distributed, 338 students completed the survey with a response rate of 84.5%. Overall, surgery (24%) and internal medicine (20%) were the most favored, specialties with only 5% of the students selecting pathology as their first choice. Patient-doctor interaction (72.2%) was perceived as the most important factor in not choosing pathology as a future career. Taking an elective course, younger age groups, and year in medical school were all significantly associated (p<0.001) with an interest in pathology.

Conclusion

In this study, most of the students indicated surgery and internal medicine as their first specialty choices. Only 5% of the students chose pathology as their first choice. Two-thirds of medical students perceived pathologists do not interact with patients. A significant association was found between younger age groups, enrolling for a pathology course, and having an interest in pathology.

## Introduction

One of the most important decisions any medical student takes after graduation is to select a specialty that would stay with the rest of his career. During undergraduate medical education, most students develop an interest in some of the specialties they encounter during their clinical rotation. Pathology being invisible in students’ day-to-day clinical rotations and their lack of understanding of its role in patient care may affect the popularity of pathology as a viable choice [1]. As a result, the number of senior medical students choosing pathology as their future career has been in decline in the last few decades [2]. A growing body of literature shows that there are multiple factors that may influence medical students' preferences in specialty selection, one of which being patient contact, as pathologists work in secluded laboratories [3]. Moreover, gender and marital status were found to have an effect on females’ career choices while personal preference and work achievement were crucial among males [4-5].

Many attempts have been made to increase the number of medical graduates choosing pathology as a residency program. In achieving this goal, some institutions implemented specific programs to give medical students an opportunity to explore pathology as basic science and influence their future residence choices [6-7]. Despite these efforts, the shortage in recruiting residents into pathology persisted [7]. A study in Australia reported only 3% of medical students chose pathology as one of their top three choices [8]. Another study stated that only 6% of their students have favored immunology, a subspecialty of pathology, as a specialty of choice [9]. A similar study conducted in Kuwait also showed that only one out of 144 students chose histopathology as a future career [10].

In Saudi Arabia, a number of studies have been published that looked at medical students’ career choices. A study conducted in Dammam included 651 medical students and found that internal medicine was the preferred specialty for 56 (14.8%) students followed by family medicine (35; 9.2%) and only seven (1.1%) students chose pathology as their future career [11]. Among other studies conducted in Saudi Arabia for future specialty choice, pathology was not one of the selected choices [12-13]. A recent study in Dhahran stated that 22 (8.7%) of the 254 who responded to the survey indicated that they would consider pathology as a future career; only two (0.7%) indicated that they make it their first choice [14].

Despite the shortage in the number of pathology specialists in Saudi Arabia, less attention was given to the factors that led to such a low number of pathologists. Therefore, this study aimed to investigate the perceptions and determinants of medical students’ preferences towards pathology as a future career in three medical colleges in Riyadh, Saudi Arabia.

## Materials and methods

This is a cross-sectional study in which students from three medical colleges in Riyadh, Saudi Arabia, have been invited to take part. Although the focus of this study was to assess the medical students' preferences towards pathology as a future specialty, some questions were included to determine students’ currents career preferences. This study included medical students from King Saud bin Abdulaziz University for Health Sciences (KSAU-HS), King Saud University (KSU), and Princess Nora University (PNU). The total number of students from the three universities was estimated to be 3000. With a 95% confidence level, response distribution of 10%, and margin of error of 3%, the recommended sample size of this study was 341. However, 400 questionnaires were distributed to accommodate for the non-response. Using the quota sampling technique, students were proportionally recruited from the three universities based on the number of enrolled medical students. This study recruited pre-clinical and clinical students with 172 students from KSU, 135 students from KSAU-HS, and 31 students from PNU.

A self-administered questionnaire was constructed for this study. A panel of experts consisting of medical educators, pathologists, and a biostatistician assessed the face and content validity to check the relevance and appropriateness of the items in each domain. Upon completion of the pilot, the reliability of the questionnaire was tested with an overall Cronbach's alpha of 0.6. The first part of the questionnaire addressed the demographic characteristics of the study participants such as age and gender, year of study in their respective universities, and cumulative grade point average (cGPA). The second part contained 10 questions that assessed the factors that influence students’ choice toward their future specialty. In the third part, students were asked to rank the first three future specialties they wish to pursue after medical school. The fourth part of the questionnaire included six questions that addressed students' perception of pathology as a career. Data collected were analyzed using Statistical Product and Service Solutions (SPSS) Statistics for Windows, Version 25.0 (Armonk, NY: IBM Corp). Frequencies and percentages were presented for categorical variables while mean and standard deviation were calculated for numerical variables. A chi-square test was used to assess the associations between students’ demographic characteristics with their interest in selecting pathology as a career choice. A test was declared significant if the p-value is <0.05.

## Results

Out of the 400 questionnaires distributed, 338 students completed the survey, with a response rate of 84.5%. The distribution of students in terms of their medical schools was 135 (39.9%) in KSAU-HS, 31 (9.2%) in PNU, and 172 (53.0%) in KSU (Table 1). Just over half of the students (179; 53%) were males and those aged 21 years were more (101; 31.1%) compared to other age groups. Around one-third of the students were in the fourth year of their medical education and close to half of the students (162; 47.9%) reported that their cGPA was above 4.5.

**Table 1:** Demographic characteristics of medical students KSAU-HS: King Saud bin Abdulaziz University for Health Sciences; PNU: Princess Nora University; KSU: King Saud University; cGPA: cumulative grade point average

Variables	Categories	N	%
Medical School	KSAU-HS	135	39.9
	PNU	31	9.2
	KSU	172	50.9
Gender	Male	179	53.0
	Female	159	47.0
Age (years)	19 - 20	67	20.6
	21	101	31.1
	22	72	22.2
	23+	85	26.2
cGPA	< 4.0	59	17.5
	4-4.5	117	34.6
	>4.5	162	47.9
Year in Medical School	First	16	4.7
	Second	48	14.2
	Third	57	16.9
	Fourth	117	34.6
	Fifth	57	16.9
	Sixth	43	12.7

**Figure 1:**
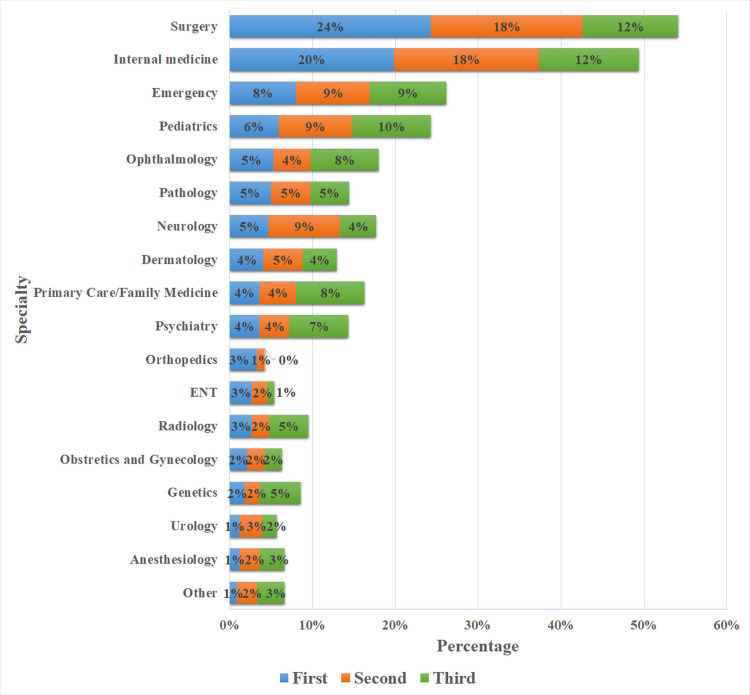
First, second, and third specialty choices among medical students

Figure 1 shows the first, second, and third specialty choices among the medical students. Surgery was the most favorable first specialty, with 24% followed by internal medicine and emergency medicine with 20% and 8%, respectively, and only 5% of the students selected pathology as their first choice. Furthermore, the number of students who selected surgery (18% and 12%) and internal medicine (18% and 12%) as their second and third choice were more when compared to all other specialties. Students who selected pediatrics (10%), ophthalmology (8%), family medicine (8%), psychiatry (7%), and genetics (5%) as their third choices were more when compared to those who selected them as their first choice. Neurology was the only specialty where students’ choice peaked in their second preference and then declined in the third.

**Figure 2:**
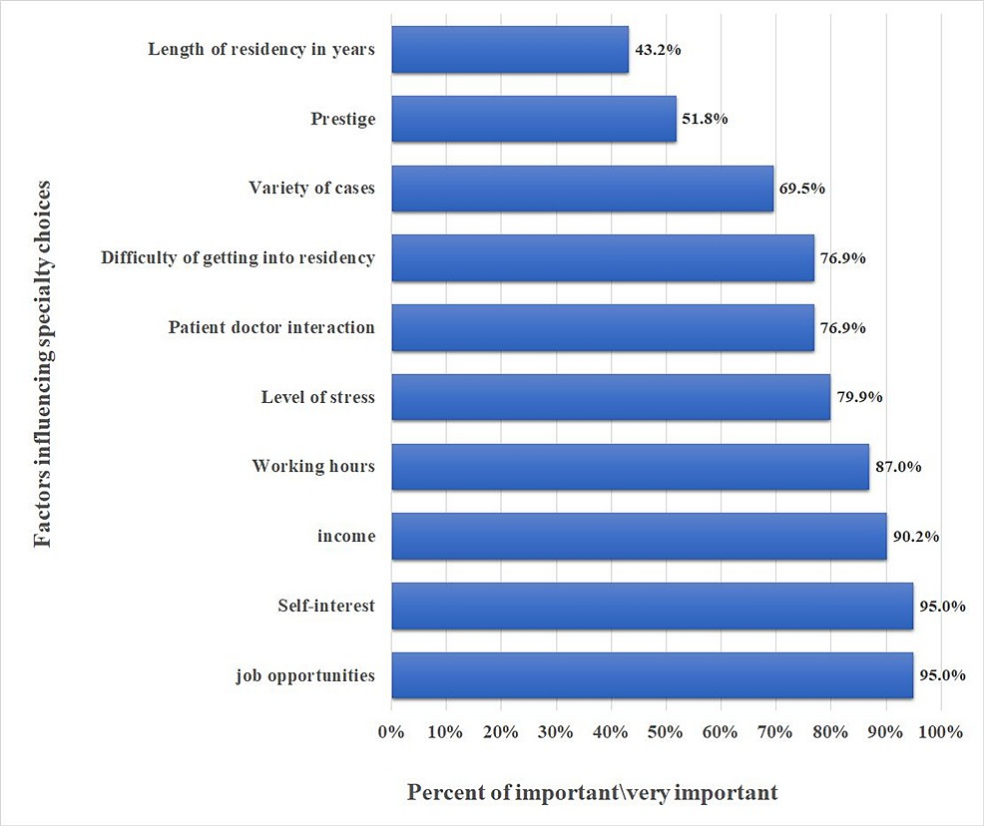
Factors influencing specialty choices among medical students

Figure 2 shows the findings of the factors influencing specialty choices among the students from the three data collection sites. Job opportunities and self-interest were the most important factors in choosing a future career, which was reported by 95% of the students. Other factors that students mentioned to be important were income (90%), working hours (87%), level of stress involved in the specialty (79.9%), and patient-doctor interactions (76.9%). On the other hand, the length of the residency program (43.2%) was the least important factor in determining their specialty choices.

**Figure 3:**
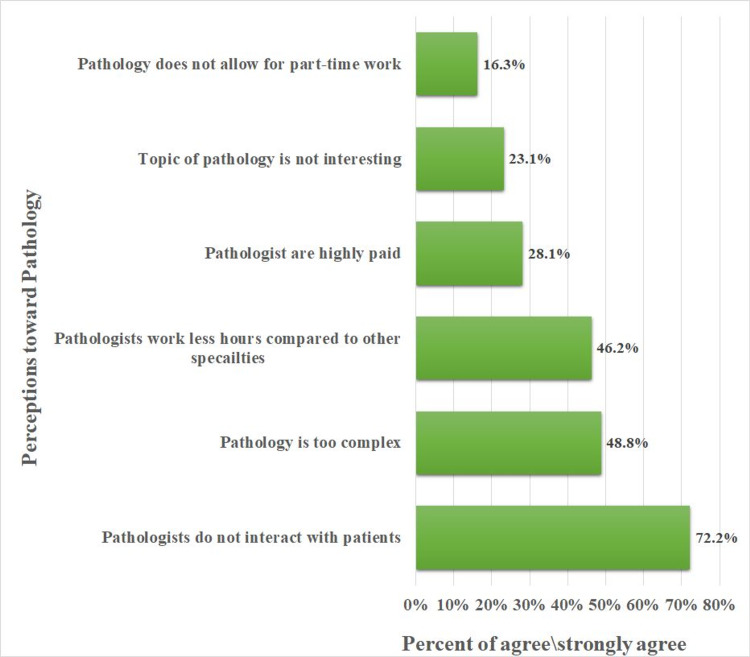
Medical students’ perceptions of pathology as a future career

Figure 3 described the students’ perception of pathology as a specialty. Seventy-two percent of the students perceived that pathologists do not interact with patients. Around half of the students think that pathology is too complex while 46.2% of the students think that pathologists work fewer hours than other specialties.

Table 2 shows the factors associated with students’ interest in pursuing pathology as a career. Students who enrolled in a pathology elective were more likely to be interested in pathology compared to those who didn’t (44% vs 16.3%; P-value <0.001). Younger students were more interested in pathology when compared to older students with a P-value of <0.001. Interest in pathology diminished as students progressed through to the clinical phases of their education. No significant associations were recorded between gender, cGPA, type of medical school attended, and students’ interest in pathology.

**Table 2:** Factors associated with students’ interest in choosing pathology as a future career cGPA: cumulative grade point average

		Interested in pathology						p-value
		Yes		No		Not sure		
		N	%	N	%	N	%	
Have you ever enrolled in a pathology elective?	Yes	11	44.0	6	24.0	8	32.0	<0.001
	No	51	16.3	161	51.4	101	32.3	
Medical school	KSAU-HS	31	23.0	57	42.2	47	34.8	0.149
	PNU	7	22.6	15	48.4	9	29.0	
	KSU	24	14.0	95	55.2	53	30.8	
Gender	Male	34	19.0	84	46.9	61	34.1	0.62
	Female	28	17.6	83	52.2	48	30.2	
Age (years)	19 - 20	18	26.9	17	25.4	32	47.8	<0.001
	21	14	13.9	52	51.5	35	34.7	
	22	16	22.2	40	55.6	16	22.2	
	23+	10	11.8	53	62.4	22	25.9	
cGPA	< 4.0	6	10.2	33	55.9	20	33.9	0.388
	4-4.5	22	18.8	60	51.3	35	29.9	
	>4.5	34	21.0	74	45.7	54	33.3	
Year in medical school	first	7	43.8	5	31.3	4	25.0	<0.001
	second	13	27.1	8	16.7	27	56.3	
	third	10	17.5	29	50.9	18	31.6	
	fourth	17	14.5	57	48.7	43	36.8	
	fifth	10	17.5	38	66.7	9	15.8	
	Sixth	5	11.6	30	69.8	8	18.6	

Table 3 compared the first choice specialty between males and females of the medical students. Overall, no significant gender differences in the first preference specialties were observed in our study (P-value = 0.408). However, with regard to their first choice specialty, females were more interested in obstetrics and gynecology compared to males (3.8% vs 0.6%; P-value = 0.041).

**Table 3:** Differences between male and female medical students with regard to first specialty preference

1st Preference	Gender				
	Male		Female		
	N	%	N	%	p-value
General surgery	48	26.8	34	21.4	0.248
Internal medicine	39	21.8	28	17.6	0.334
Dermatology	8	4.5	6	3.8	0.748
Ophthalmology	6	3.4	12	7.5	0.094
Emergency	17	9.5	10	6.3	0.279
Pediatrics	13	7.3	7	4.4	0.26
Pathology	6	3.4	11	6.9	0.142
Psychiatry	7	3.9	5	3.1	0.691
Radiology	4	2.2	5	3.1	0.605
Anesthesiology	2	1.1	2	1.3	0.866
Genetics	3	1.7	3	1.9	0.89
Primary Care/Family Medicine	5	2.8	7	4.4	0.428
Urology	3	1.7	1	0.6	0.351
Neurology	7	3.9	9	5.7	0.438
Orthopedics	4	2.2	7	4.4	0.254
Obstetrics and Gynecology	1	0.6	6	3.8	0.041
ENT	4	2.2	5	3.1	0.605
Other	2	1.1	1	0.6	0.62

## Discussion

The main aim of this study was to identify the reasons for the lower uptake of pathology residency and investigate the determinants of medical students’ preferences towards pathology as a future career in three medical colleges in Riyadh, Saudi Arabia. In this study, general surgery and internal medicine were found to be the most favorable career choices while anesthesiology and urology were reported as the least favorable. Our findings are similar to studies conducted in Jordan [1], Pakistan [15], Ethiopia [16], and Saudi Arabia [12,17], which also found that the majority of the students have selected surgery followed by internal medicine as their future career. Although pathology was ranked as the sixth highest specialty, only 5% of the medical students indicated that they would make pathology their first choice.

Overall, no significant gender differences in the first preference specialties were observed in our study. However, when the relationship between gender and individual subjects was examined, only obstetrics and gynecology showed a higher percentage of females made this specialty their first choice. This study’s finding is consistent with French and Indian studies that showed women were more likely to prefer obstetrics and gynecology as their first and second [18-19]. Numerous studies have also reported that males traditionally choose surgery [1,15-16,20]. In contrast, our findings show that there is no significant difference between males and females in terms of their preference for general surgery. However, our finding agreed with another study conducted in Dammam, Saudi Arabia [11], which found that both genders did not differ in their general surgery choices.

Study participants were asked about the factors they thought would influence their career selection. Job opportunities, interest in the specialty, income, and working hours were the highest scoring determinants in their choices. Close to three-quarters of the participants have indicated level of stress, patient-doctor interaction, and difficulty in getting into a particular residency were influential factors in specialty selections. Although ranked lowly together with the length of training, half of the participants have reported prestige as an influential factor in their decision. These findings are consistent with earlier studies that highlighted similar motivational factors [8,10-11,21-22].

Participants were asked about their perception of pathology and the majority of the students perceive that pathologists do not interact with patients. This perception partly explains the low number of students choosing pathology as their first choice. A variety of reasons were put forward that may further explain the low uptake of pathology among medical students. One of the widely reported factors is the minimal exposure of students to practical pathology during their medical school years [23-24]. In a medical school in North Central Nigeria, one study reported that time allocated for the pathology course during undergraduate education may play a negative role in selecting pathology as a career [25]. Our study finding also agrees with a Pakistani study that reported that students favored better integration of pathology into the curriculum [26]. A Canadian study found that non-pathology clinical residents indicated that misconceptions and stereotypes about pathology took them away from selecting pathology as a career. On the contrary, pathology residents found the specialty interesting and full of joy when correct diagnoses are made [18].

In the present study, we investigated factors that are associated with students’ interest in choosing pathology as a future career. Enrolment in a pathology course during the medical school years was significantly associated with choosing pathology. Further analyses showed that higher percentages of younger students and those in the first two years of medical school are more interested in pathology than the senior medical students. The invisibility of pathology from the curriculum beyond the second year of undergraduate medical education and the lack of public knowledge about pathology was reported to may have an influence on the popularity of pathology [27-28]. To counter the ever-diminishing interest in pathology, many universities around the world have created post-second-year fellowships or intercalating years where students are given the opportunity to explore more courses in pathology to increase the uptake of pathology as a future career. Harnessing the interest in pathology, a study in the US found that exposing medical students to a well-structured pathology course early in their medical education would, upon graduation, enhance the number of students choosing the profession [29]. Another qualitative study in India suggested some modifications to the way pathology is taught at an undergraduate level and including the pathology in the interns’ rotations may improve the perceptions of medical students towards pathology as a career [30]. Despite all these efforts to increase the number of students choosing pathology, some universities have significantly reduced the teaching and practical hours thereby affecting the perception and the motivation of future pathology residents [14].

Limitations

Despite the comprehensive methodology and design in this study, certain limitations need to be considered. First, the data were collected from public medical schools, and no private medical colleges were included thereby limiting the generalizability of the results. Second, the relationship between enrolment in a pathology elective and interest in the specialty could be influenced by the students’ pre-existing interest in pathology. Finally, this cross-sectional self-administered study only measured students’ interest in pathology but not whether this will be their firm choice when they graduate.

## Conclusions

In this study, the majority of the students placed surgery and internal medicine as their first specialty choices with 5% choosing pathology as their future career. As for the factors influencing career choices, our findings show that job opportunities and self-interest were the most important factors in choosing a future career followed by the level of income. Two-thirds of the students perceived that pathologists do not interact with patients while close to half of them thought pathology is too complex to understand. Enrolment in a pathology elective and being in the pre-clinical years were positively associated with greater interest in pathology. This multi-institutional study highlighted the negative perceptions and probable factors that influence medical students’ choice of pathology as a career. However, further research is a need to retrospectively explore the perceptions of current pathology residents and the reasons that encouraged them to choose pathology as a career.
